# Introducing an accountability framework for polio eradication in Ethiopia: results from the first year of implementation 2014-2015

**DOI:** 10.11604/pamj.supp.2017.27.2.10939

**Published:** 2017-06-09

**Authors:** Aron Kassahun, Fiona Braka, Kathleen Gallagher, Aregai Wolde Gebriel, Peter Nsubuga, Pierre M’pele-Kilebou

**Affiliations:** 1 World Health Organization Country Office, Addis Ababa, Ethiopia; 2 Centers for Disease Control and Prevention, Global Immunization Division, Atlanta, GA, USA; 3 Global Public Health Solutions, Atlanta, GA, USA

**Keywords:** Accountability framework, polio eradication, staff performance, program performance indicators

## Abstract

**Introduction:**

the World Health Organization (WHO), Ethiopia country office, introduced an accountability framework into its Polio Eradication Program in 2014 with the aim of improving the program's performance. Our study aims to evaluate staff performance and key program indicators following the introduction of the accountability framework.

**Methods:**

the impact of the WHO accountability framework was reviewed after its first year of implementation from June 2014 to June 2015. We analyzed selected program and staff performance indicators associated with acute flaccid paralysis (AFP) surveillance from a database available at WHO. Data on managerial actions taken were also reviewed. Performance of a total of 38 staff was evaluated during our review.

**Results:**

our review of results for the first four quarters of implementation of the polio eradication accountability framework showed improvement both at the program and individual level when compared with the previous year. Managerial actions taken during the study period based on the results from the monitoring tool included eleven written acknowledgments, six discussions regarding performance improvement, six rotations of staff, four written first-warning letters and nine non-renewal of contracts.

**Conclusion:**

the introduction of the accountability framework resulted in improvement in staff performance and overall program indicators for AFP surveillance.

## Introduction

Accountability is the ownership of responsibilities combined with the obligation to report on the discharge of those responsibilities [1]. An accountability framework makes roles, responsibilities and expectations clear, and supports the availability of reliable and timely reports about intended and actual results [2]. The framework further delineates performance measures to ensure the efficient, effective and transparent management of resources for its mandated objectives [3]. Accountability has always been embedded in the structure of WHO and its operational policies and procedures. However, in response to the evolving environment of reform that constitutes accountability and transparency as core principles, coupled with the declaration of polio eradication as a public health emergency of international concern in 2014, the 2006 WHO Accountability Framework was revised. The revised WHO Accountability Framework is designed to support the Organization's results-based management approach whereby delegated responsibility, authority, and accountability exist in a decentralized environment at all levels of the Organization, and to underline its commitment to the shared values and culture of accountability and transparency [4]. The Polio Eradication Initiative was launched in 1988 and has since then recorded substantial achievements in kicking polio out of the African continent. Despite the encouraging gains, the initiative has faced several setbacks over the years. One of the setbacks, specifically in the Horn of Africa (HOA), was the importation of wild poliovirus (WPV) into Somalia in mid-2013, which then spread to Kenya and Ethiopia. A total of 223 confirmed WPV type 1 cases were reported from the HOA; of these 199 cases were from Somalia, 14 were from Kenya and 10 were from Ethiopia. During this time, efforts to introduce an accountability framework into the existing monitoring and evaluation system of the WHO Ethiopia's Expanded Program on Immunization (EPI) program had started, and the importation of the poliovirus in mid-2013 provided an opportunity to fast track the activity, considering the increase in technical surge capacity in the country, particularly in the affected Somali Region. Following the confirmation of the first WPV in Somalia, response activities were initiated that included supplemental immunization activities (SIAs), and intensified surveillance. The response necessitated an increase in WHO presence in the field in order to rapidly interrupt poliovirus transmission. By the end of 2014, a total of 75 “surge” staff had been recruited and deployed in Ethiopia, mainly to the Somali Region. A monitoring and accountability mechanism to track performance of the newly-recruited and existing staff become more critical to improve performance and ultimately reduce the risks of further transmission of the virus.

The EPI team in the WHO country office, totaling 113 in 2014-2015, is deployed at central, regional and zonal levels, in alignment with the country's administrative levels. Regional coordinators, delegated and present in the 9 regions and 2 administrative cities, coordinate and link the field team with the central team that is organized into three technical subunits: Surveillance, Routine Immunization and Monitoring and Evaluation (M&E). The staffing in Somali Region was uniquely structured following the WPV1 importation, with officers present at the lowest administrative level (woreda), reporting to zonal coordinators who then reported to the regional coordinator. The Accountability Framework tool was adopted from the global framework in 2014 with key principles that included better accountability as an organization to the outcomes WHO is committed to delivering [4]. Although the M&E unit at the WHO country office had a system where monthly reports from field officers were submitted and feedback regularly given, there was no systematic way of monitoring other expected program deliverables, and the accountability component was missing. We describe the process and initial results of implementing an accountability framework to guide the management of the WHO Ethiopia EPI program and improve performance of staff.

## Methods

**Developing the accountability framework:** a review of the Standard Operating Procedures (SOPs) for EPI staff at WHO Ethiopia was conducted to identify deliverables at different levels. Additionally, the Terms of References, (TORs) for the central team, field officers and zonal and woreda facilitators were reviewed and a list of key deliverables to be monitored at the individual officer level was determined. A total of 17 deliverables were selected. Based on the SOPs and TORs at different levels, the deliverables were grouped into themes: routine immunization, SIAs, M&E and program management (Table 1). The next step was to identify reporting timelines, quality indicators and monitoring levels. The timeline of reporting for each deliverable was set for submission to the regional and central level. Monthly deliverables were to be submitted to regional/zonal coordinators before the 7th day of the following month and before the 15th to the central level. Quarterly deliverables were expected to be delivered at regional level before the 15th of the following month and before the end of the same month at the central level. The same timeline of reporting was applied for biannual and annual set of deliverables, but SIA and outbreak deliverables were ad hoc and expected whenever there was a campaign and outbreak in a given area. The deliverables submission was monitored both at the regional and central level, and a template to track timelines by the individual officers was introduced at the regional level. Besides timeliness, the tool monitors quality of submitted deliverables using selected indicators, which the regional Coordinator compiles and shares with central level. The quality monitoring indicators and the respective score is presented in Table 2. The quality monitoring template and scoring was done through a consultative process between central and field officers in late 2013. Finally we developed a template and generate a summary dashboard showing performance by individual officer by quarter that is shared among the team at the end of each quarter.

**Table 1:** List of deliverables categorized by program thematic areas, WHO Ethiopia, 2014-2015

Surveillance	Routine Immunization (RI)	SIA	Monitoring and Evaluation	Program Management
Proportion of Validated AFP Cases	Zone and Woreda level RI data submission (quarterly)	Pre-campaign, intra and post assessment	Annual basic data submission	Annual work plan
Timeliness of 60 days follow up	RI prioritization (quarterly)	Submission of admin coverage data	Timeliness of Personal Digital Assistant (PDA) data submission (monthly)	Monthly activity report
Non-polio AFP (NPFAP) rate		Submission of IM data	Biannual Data Quality Self-Assessment (DQS) (bi-annually)	Quarterly work plan update
Stool adequacy rate		Submission of Rapid Convenient Survey (RCS) data	Outbreak investigation report	
Contact sampling (High-risk areas)				
Polio (quarterly ); Measles (bi-annually) ; Neonatal Tetanus (NNT) (annual) Risk Assessment				
# HFs visited monthly				

**Table 2:** Monthly activity report quality monitoring indicators, WHO Ethiopia, 2014-2015

INDICATOR	DEFINITION	Score
Use of previous feedback	Whether action is taken for the previous month, feedback given by the Central Team	**15**
Consistency	No conflicting information, data, etc.	**15**
Use of weekly update	If data from weekly update is used for subsequent planning and actions	**10**
Use of PDA data	Whether data/information from PDA is used for reporting and subsequent planning and actions	**40**
Use of standard reporting format	If standard format is used to report	**10**
Completeness	Filling out all the components of the reporting format	**10**

**Study population:** a total of 38 staff were studied. All WHO field officers namely regional coordinators, zonal officers and woreda officers, were included in the accountability framework.

**Data collection:** we designed a relational database in Microsoft Access, at the central level that systematically captured all submitted deliverables. All deliverables are shared electronically, and the information is captured from a central mailbox that was created exclusively for this purpose and collated into the database. The system has embedded dates of expected submission and classifies individual deliverables accordingly. The database is also linked to the polio surveillance database whereby surveillance and related staff performance indicators are generated at the end of each quarter. The deliverables were reviewed for a twelve month period: July 2014 - June 2015.

### Definitions

**AFP case validation:** reported AFP cases are expected to be validated by WHO field officers. The validation includes confirming if the case is truly acute flaccid paralysis and if the information included on the case investigation form is accurate. Each officer is expected to validate at least 80% of all reported cases in his/her catchment area.

**Late AFP case:** an AFP case is classified as “late” when a) the first stool is collected more than 14 days after onset of paralysis and/or b) when the time between 1st and 2nd stool collection is more than 24 hours and/or c) the stool condition is bad, based on the amount of stool collected and temperature at receipt of specimen, as evaluated by a laboratory technician.

**60-day follow up:** follow- up assessments of late AFP cases are done my WHO officers 60 days after the onset of paralysis. Officers are expected to complete follow-up reports for 100% of late AFP cases. In addition to calculating the proportion of late AFP cases with 60 days follow-up report, we analyze timeliness of follow-up reports, for those late cases with follow-up reports, using time between date of onset of paralysis and date when follow-up was done. According to the national guideline, all follow-up of late cases should be done before 90 days of onset of paralysis.

**Non polio AFP (NPAFP) rate:** a rate of reported AFP case for the under 15 years of age population in a defined geographical unit within 12 months. Woreda, the third geographic unit, is used to calculate the rate. A minimum of 2 AFP cases are expected to be reported for 100,000 under 15 years population in 12 months time.

**Stool adequacy rate:** this is calculated based on the number of reported AFP cases which are not classified as late cases divided by the total number of reported cases within 12 months in a geographic unit. The indicator is calculated starting at Woreda level up to regional level and the minimum target is 80%.

## Results

The trend in number of regions meeting the minimum proportion of all AFP cases validated by Field Officers increased from five regions in 2013( Amhara, Benishangul-Gumuz, Oromia, SNNPR and Tigray) to seven Regions in 2014 to all regions (except Hareri) in 2015. The proportion of validated AFP cases in Somali Region increased from 39% in 2013 to 86% in June 2015 (Table 3). The proportion of late AFP cases with 60-day follow-up done and report submitted nationally was 83% in 2013, 77% in 2014 and 58% in 2015. We disaggregated the proportion by regions and found that Somali region was 62% in 2013 and reached 84% in 2015 (Table 3). Furthermore, the proportion of 60 day follow-ups done within 90 days of onset of paralysis was 67% in 2013, 69% in 2014 and 88% in 2015.

**Table 3:** Validation of reported AFP cases by Region by year: 2013 - June 2015, WHO Ethiopia

	2013			2014			Jan-June 2015		
	% AFP cases Validated	% FU Late AFP cases Done	% FU Done 60-90 Days	% AFP cases Validated	% FU Late AFP cases Done	% FU Done 60-90 Days	% AFP cases Validated	% FU Late AFP cases Done	% FU Done 60-90 Days
**ADDIS ABABA**	21%	100%	0%	52%	100%	0%	100%	100%	100%
**AFAR**	54%	100%	33%	70%	75%	33%	100%	NA	NA
**AMHARA**	81%	89%	73%	80%	69%	72%	93%	56%	90%
**B/GUMUZ**	93%	86%	100%	85%	100%	67%	86%	0%	NA
**DIRE DAWA**	71%	NA	NA	0%	NA	NA	100%	NA	NA
**GAMBELLA**	63%	100%	0%	92%	100%	25%	100%	NA	NA
**HARERI**	100%	0%	NA	33%	100%	0%	50%	0%	NA
**OROMIA**	74%	85%	71%	81%	71%	75%	86%	63%	95%
**SNNPR**	83%	85%	75%	82%	85%	94%	83%	50%	33%
**SOMALI**	39%	62%	29%	83%	84%	66%	86%	50%	80%
**TIGRAY**	93%	91%	60%	100%	83%	60%	89%	100%	100%
**NATIONAL**	**74%**	**83%**	**67%**	**81%**	**77%**	**69%**	**88%**	**58%**	**88%**

Data Source: National AFP Surveillance, WHO Ethiopia

**Figure 1:**
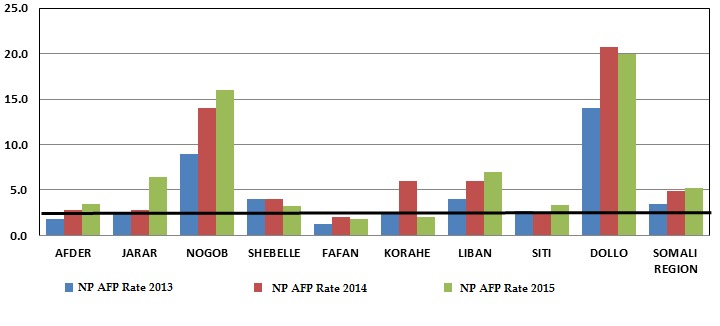
Trend in non-polio AFP Rate by zone, Somali Region, Ethiopia 2013 – June 2015

When we analyzed selected program performance indicators, NPAFP and stool adequacy rates, the NPAFP rate at the national level was at 2.7 cases per 100,000 children under 15 years old in 2013, 3.1/100,000 in 2014 and 3.2/100,000 in 2015; which is above the target of 2.0/ 100,000 children under 15 years old. There were three regions, Addis Ababa, Afar and Tigray regions, with NPAFP below the expected standard in 2013 while all regions achieved the expected rate in 2014 and maintained this through June 2015 (except Tigray). The stool adequacy rate at the national level increased from 88% in 2013 to 93% in 2015. Three regions did not achieve the minimum target of 80% in 2013 and 2014 but by 2015 all regions had achieved the 80% target (Table 4). We analyzed program indicators in Somali region by zone, and identified that Nogob and Dollo zones performed above the expected rate throughout the study period: the NPAFP rate in Dollo zone was 14.0/100,000 at the end of 2013 and increased to 20.7/100,000 in 2014, and the rate is at 20.0/100,000 as of June 2015. In Nogob zone, the NPAFP rate was at 9.0/100,000 in 2013, 14.0/100,000 in 2014 and 16.0/100,000 as of June 2015. All zones achieved the minimum target 0f 2.0/100,000 except Fafan zone, in 2013 and 2015, and Afder in 2013 (Figure 1).

**Table 4:** AFP surveillance performance indicators by region, 2013- June 2015, Ethiopia

	2013			2014			Jun-15		
REGION	NPAFP Per 100,000	Stool Adq (%)	Achieved Indicators	NPAFP per 100,000	Stool Adq(%)	Achieved Indicators	NPAFP per 100,000	Stool Adq (%)	Achieved Indicators
**ADDIS ABABA**	1.6	91	One indicator	3.0	86	Both Indicators	2.9	80	Both indicators
**AFAR**	1.8	100	One indicator	3.8	87	Both Indicators	5.0	100	Both indicators
**AMHARA**	2.9	88	Both Indicators	2.7	86	Both Indicators	3.6	92	Both indicators
**BENISHANGUL**	3.0	69	One indicator	4.4	85	Both Indicators	3.1	86	Both indicators
**DIRE DAWA**	3.0	100	Both Indicators	2.0	100	Both Indicators	2.0	100	Both indicators
**GAMBELLA**	2.8	100	Both Indicators	2.0	69	One Indicator	2.0	100	Both indicators
**HARERI**	2.0	50	One indicator	5.0	67	One Indicator	4.0	100	Both indicators
**OROMIA**	2.7	89	Both Indicators	3.1	88	Both Indicators	3.1	91	Both indicators
**SNNPR**	2.7	92	Both Indicators	2.9	92	Both Indicators	2.7	97	Both indicators
**SOMALI**	3.4	64	One indicator	4.9	74	One Indicator	5.2	93	Both indicators
**TIGRAY**	1.9	86	One indicator	2.6	90	Both Indicators	1.6	81	One Indicator
**NATIONAL**	2.7	88	Both Indicators	3.1	87	Both Indicators	3.2	93	Both indicators

Data Source: National AFP Surveillance, WHO Ethiopia

**Figure 2:**
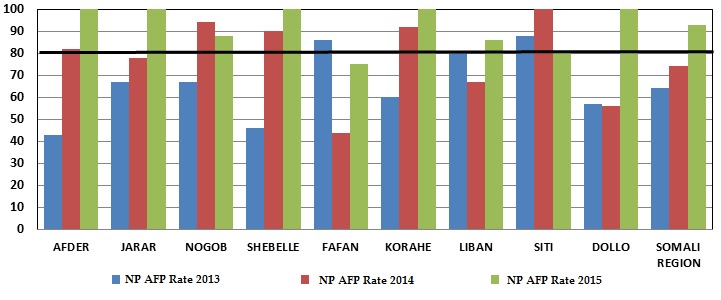
Trend in stool adequacy rate by zone, Somali Region, Ethiopia, 2013 – June 2015

Only three zones in Somali region achieved the stool adequacy target in 2013 and this increased to five zones in 2014. All zones but Fafan achieved the target in 2015 (Figure 2). Since the start of the monitoring and accountability system, the following managerial actions were taken based on the performance dashboard: 11 written acknowledgments to good performing staff , six discussions for improvement, six rotation of staff, four written first warning letters, and nine non-renewal of contract for poor performing staff were issued or implemented respectively.

## Discussion

We found that instituting the accountability framework into the existing monitoring and evaluation system for the WHO Ethiopia EPI team contributed to improved staff performance. We also found out that there was an overall improvement in key program performance indicators during the study period.

The proportion of validated AFP cases significantly improved both at the national and regional levels following the introduction of the monitoring system; chronically low-performing regions including Somali, Gambella and Afar have shown good improvement. A similar trend was observed in the proportion of late AFP cases with follow-up report submitted within 90 days of onset of paralysis from only 67% of the cases with follow-up reports in 2013 to 88% at the end of June 2015.This study highlighted the improvement in program indicators, NPAFP and stool adequacy rate, from only six regions achieving the minimum target for both indicators in 2013 to all regions except Tigray achieving both targets in 2015. Instituting accountability into health programs is becoming increasing popular, according to a study by O'Hagan et al. Healthcare providers are constantly striving to improve quality and efficiency by using performance management systems and quality improvement initiatives. Creating and maintaining a culture of accountability are important for achieving this end because accountability is the reason for measuring and improving performance [5].

Our findings on the impact of the accountability framework on performance are similar to that observed in Nigeria, where the WHO country office has been implementing a systematic accountability framework to improve performance of the polio eradication program. According to Tegegne et al [6], a significant change in process indicators of both AFP surveillance and routine immunization was demonstrated over the first year of implementation of the accountability framework. The WHO Representative in Nigeria attributed the progress in interruption of WPV transmission in Nigeria to the institutionalization of accountability framework by the government and partners to ensure those personnel delivered as expected with appropriate actions being taken based on performance, among other key contributing factors [7].

Various United Nation organizations have used different types of accountability frameworks. According to a joint inspection report by M.Mounir Zahran et al, United Nations organizations possess a stand-alone formal accountability framework (seven United Nations Organizations). Three secretariat entities possess a program level accountability framework. Other United Nations system organizations have various key components of accountability to varying degrees, several of them with strong internal control systems or components in place [8]. As an example, the UNICEF accountability framework highlights key functional elements of staff and management accountability at all levels of the organization. These basic functional elements are articulated in roles, responsibilities, and processes outlined in office-specific management plans and individual job descriptions [9]. Accountability frameworks are also becoming popular in nongovernmental organizations (NGOs). A study by Cunningham et al. at Oxfam Ireland argues that imposed accountability frameworks increase accountability towards donors and stakeholders, but do not increase accountability towards beneficiaries. However, the authors note that through the application of the adaptation measures outlined within their report, NGOs can balance each level of accountability, which can lead to an overall increase in program impacts towards beneficiaries [10].

We cannot conclude that the introduction of the accountability framework is solely responsible for improvement in program indicators where increased human resource in Somali Region, government focus to an outbreak that could enhance surveillance and other contributing factors interplay with performance. However, the improvement in individual level performance can be attributed to the transparent and performance monitoring framework instituted into the monitoring and evaluation system of the country office. Improved accountability is often called for as an element in improving health system performance. At first glance, the notion of better accountability seems straightforward, but it contains a high degree of complexity. For accountability to serve effectively as an organizing principle for health systems reform, conceptual and analytical clarity is required [11].

## Conclusion

In conclusion, introducing an accountability framework that is evidence-based to enhance staff performance and increase transparency, in programs such as polio eradication, was very useful and effective in improving performance towards meeting program targets in WHO Ethiopia. The accountability framework should be maintained and further strengthened to incorporate other aspects of the immunization program towards achievement of eradication and elimination targets. We further recommend expanding the accountability framework to other country programs to contribute to progressive staff and organizational performance improvement.

### What is known about this topic

- An accountability framework in polio eradication has already been implemented in Nigeria: one of the three polio endemic countries in the world. In the published article by the team, the monitoring framework is believed to have brought an impact and is gearing the program closer to eradication;
- Recently the African Regional office, WHO AFRO, has instituted a similar accountability framework tool to monitor program performance at country level. This is driven based on the experience at country level of Nigeria and Ethiopia and the positive outcomes of the monitoring tool.

### What this study adds

- The study provides additional evidence of positive impact from similar monitoring systems. Clearly identifying deliverables and continuously monitoring performance at the individual level is very important in eradicating diseases like Polio;
- The study could be a reference for other public health problems in similar context, including elimination of measles and rubella. Such a study should be documented as part of the Polio legacy to benefit other programs.

## Competing interests

The authors declare no competing interests. The views expressed in the perspective articles are those of the authors alone and do not necessarily represent the views, decisions or policies of the institutions with which they are affiliated and the position of World Health Organization.
